# Clinical outcomes and total IgE changes with omalizumab and dupilumab in moderate-to-severe asthma: a retrospective real-world cohort study

**DOI:** 10.3389/fmed.2026.1964856

**Published:** 2026-10-20

**Authors:** Yan Chen, Boyun Yang, Wo Yao, Ran Diao, Yongfang Wang, Bohui Li, Ting Li, Liuya Ge, Yingying Hu, Huiying Wang

**Affiliations:** 1 Department of Allergy, Second Affiliated Hospital of Zhejiang University School of Medicine, Hangzhou, Zhejiang, China; 2 Outpatient Care Department, Second Affiliated Hospital of Zhejiang University School of Medicine, Hangzhou, Zhejiang, China

**Keywords:** asthma, biologic therapy, dupilumab, immunoglobulin E, omalizumab

## Abstract

**Background:**

Direct real-world comparisons of omalizumab (OMA) and dupilumab (DUP) for moderate-to-severe asthma remain limited. We compared clinical and biomarker changes during treatment with these biologics.

**Methods:**

This retrospective, single-center, real-world cohort included 72 adults who initiated OMA (*n* = 48) or DUP (*n* = 24) between January 2024 and December 2025 and continued treatment through approximately 4 months. Outcomes were assessed at baseline and approximately 4 and 12 months. Exploratory analyses compared four-month changes between groups, and available repeated measurements were evaluated using adjusted linear mixed-effects models.

**Results:**

Both OMA and DUP treatments were associated with improved asthma control and pulmonary function at 4 months, with no clear between-group differences in changes in Asthma Control Test (ACT) score, blood eosinophil count, fractional exhaled nitric oxide, or pulmonary function parameters. Adjusted models showed improvements over time in ACT score (*P* < 0.001) and percent-predicted forced expiratory volume in one second (*P* = 0.002), without significant treatment-group effects or treatment-by-time interactions. Serum total immunoglobulin E (IgE) increased with OMA and decreased with DUP at 4 months, with a significant four-month treatment-by-time interaction (*P* < 0.001) in the longitudinal model.

**Conclusion:**

In this cohort, OMA and DUP were each associated with improvements in asthma control and pulmonary function, with no clear between-group differences detected. Their differing 4-month changes in serum total IgE were consistent with differences in their pharmacodynamic effects.

## 1 Introduction

Asthma is a heterogeneous chronic inflammatory airway disease characterized by variable respiratory symptoms and reversible airflow limitation, affecting more than 300 million people worldwide (1). Type 2 (T2) inflammation, involving cytokines such as interleukin (IL)-4, IL-5, and IL-13 and immunoglobulin E (IgE)-mediated responses, is a common endotype of moderate-to-severe asthma (2, 3). Despite advances in inhaled therapies, some patients remain inadequately controlled, experiencing persistent symptoms, exacerbations, and impaired quality of life (4, 5).

Targeted biologic therapies have substantially expanded the treatment options for type 2 asthma (6). Among the available biologics, omalizumab (OMA) and dupilumab (DUP) are widely used in clinical practice. OMA binds circulating IgE and attenuates IgE-mediated allergic inflammation (7), whereas DUP blocks the IL-4 receptor *α* subunit and inhibits signaling by both IL-4 and IL-13 (8). Although both biologics have demonstrated efficacy in randomized controlled trials (RCTs), their mechanisms of action, eligibility criteria, and associated inflammatory phenotypes differ (7, 8).

In routine practice, biologic selection may be influenced by biomarker profiles, aeroallergen sensitization, treatment-relevant comorbidities, treatment accessibility, and clinical judgment (9, 10). These factors may create systematic differences between treatment groups and complicate the interpretation of non-randomized comparisons. Although recent observational studies have compared biologic therapies for severe asthma (11, 12), direct evidence between OMA and DUP remains limited, particularly in Asian populations.

Therefore, this study aimed to describe and compare four-month changes in asthma control, pulmonary function, and inflammatory biomarkers during OMA or DUP treatment in adults with moderate-to-severe asthma. Available 12-month measurements were also examined in exploratory analyses of extended follow-up.

## 2 Methods

### 2.1 Study design and participants

This retrospective, single-center, real-world cohort study consecutively included adults with moderate-to-severe asthma who initiated OMA or DUP at the Department of Allergy, The Second Affiliated Hospital, Zhejiang University School of Medicine (Hangzhou, China), between January 2024 and December 2025.

The inclusion criteria were: (1) age ≥18 years and a diagnosis of moderate-to-severe asthma according to the Global Initiative for Asthma guidelines (13); (2) required medium- to high-dose inhaled corticosteroids plus a long-acting β2-agonist, with or without a leukotriene receptor antagonist, to achieve or maintain asthma control, or had persistent asthma symptoms despite this treatment; (3) remained on the index biologic through the approximately 4-month assessment (T1; 16 ± 4 weeks); and (4) availability of ACT scores at both baseline (T0) and T1.

The exclusion criteria were: (1) clinically important coexisting respiratory diseases, including chronic obstructive pulmonary disease, bronchiectasis, or interstitial lung disease; (2) previous biologic therapy, allergen immunotherapy, or other systemic immunomodulatory treatment, except maintenance systemic corticosteroids prescribed for asthma; (3) repeatedly missed scheduled injections or a treatment interruption that prevented T1 assessment; (4) absence of baseline pulmonary function assessment; and (5) pregnancy, lactation, or active malignancy. External baseline pulmonary function reports were accepted, with quantitative indices treated as missing when numerical values could not be retrieved. Patients who subsequently switched biologics contributed only observations obtained before switching.

### 2.2 Biologic and concomitant treatments

Biologic treatment was selected by the treating physician as part of routine care, considering the inflammatory phenotype, treatment-relevant comorbidities, and treatment accessibility. The OMA dose and administration interval were determined using the approved pretreatment total IgE-body weight dosing table. All DUP-treated patients received a 600-mg loading dose followed by 300 mg every 2 weeks.

Background asthma therapy, including inhaled corticosteroids/long-acting β_2_-agonists, leukotriene receptor antagonists, long-acting muscarinic antagonists, and systemic corticosteroids, could be stepped up or down according to clinical need. Because medication adjustments were incompletely documented in the retrospective records, changes in dose and treatment intensity could not be reliably reconstructed.

Topical treatments for atopic dermatitis were permitted as part of routine care, but changes in their dose, frequency, or use were not systematically captured. Systemic immunomodulatory treatment for atopic dermatitis other than the index biologic was excluded, and none was initiated during follow-up. Maintenance systemic corticosteroid use reported in this study referred to asthma treatment.

### 2.3 Data collection and outcomes

Demographic and clinical information was obtained from electronic medical records. Baseline variables included age, sex, body mass index (BMI), asthma duration, and comorbidities, including allergic rhinitis, chronic urticaria, and atopic dermatitis. ACT score, blood eosinophil count (BEC), serum total IgE, fractional exhaled nitric oxide (FeNO), and pulmonary function indices were assessed at T0 and T1. Available measurements obtained at approximately 12 months (T2) were included in exploratory analyses of extended follow-up. Availability of every pulmonary function or biomarker measurement was not required.

All quantitative pulmonary function measurements were obtained at the study center using a MasterScreen system (Jaeger, Germany), following American Thoracic Society spirometry standards (14). Pre-bronchodilator values were used at T0, T1, and T2. Percent-predicted values were calculated using the ECSC/ERS reference equations described by Quanjer et al. (15). For four patients, external baseline reports contained only qualitative conclusions, such as normal pulmonary function; their numerical baseline indices were treated as missing and were not estimated.

Aeroallergen sensitization was defined as at least one positive serum aeroallergen-specific IgE result from recent pretreatment testing, using the laboratory positivity threshold. It was included in the adjusted models as a binary covariate (any vs. none), with unavailable test results treated as missing. This serum-based classification was distinct from the broader clinical assessment used for biologic selection, which could also incorporate historical allergy testing and exposure-related symptoms. For this study, polysensitization was defined as positive serum specific IgE results to allergens from two or more aeroallergen categories. Individual sensitization patterns were summarized descriptively.

### 2.4 Clinical overlap population

In a *post hoc* feasibility assessment, we used pretreatment information to identify patients who could reasonably have been considered candidates for either treatment. The clinical overlap population required clinically relevant aeroallergen sensitization and a total IgE-body weight combination within the approved OMA dosing table, together with at least one feature supporting DUP treatment: BEC ≥ 0.15 × 10^9^/L, FeNO at 50 mL/s (FeNO_50_) ≥ 25 ppb, or maintenance systemic corticosteroid use. Patients were not included if the available records were insufficient to confirm both the OMA criteria and at least one DUP-supporting feature. Baseline characteristics and the availability of paired follow-up data were summarized descriptively to assess the feasibility of a restricted comparison.

### 2.5 Statistical analysis

Continuous variables are presented as mean ± standard deviation or median (interquartile range), as appropriate, and categorical variables as number and percentage. Between-group baseline comparisons used Student's *t*-test or the Mann–Whitney *U*-test for continuous variables and the chi-square test or Fisher's exact test for categorical variables.

Short-term analyses were conducted separately for each outcome among patients with paired T0 and T1 measurements; completeness across all outcomes was not required. Within-group changes were evaluated using the paired *t*-test or Wilcoxon signed-rank test, as appropriate. Between-group differences in change, calculated as the T1 value minus the T0 value, were evaluated using the Mann–Whitney *U-*test. No missing outcome values were imputed.

Changes from T0 to T1 in ACT score, percent-predicted forced expiratory volume in one second (ppFEV_1_), and serum total IgE were analyzed using adjusted linear mixed-effects models. Treatment group, time as a categorical variable, and the treatment-by-time interaction were included as fixed effects, with adjustment for age, sex, atopic dermatitis, and binary aeroallergen sensitization. A patient-specific random intercept accounted for within-participant correlation. Serum total IgE was natural-log transformed because of its skewed distribution. Models were estimated using restricted maximum likelihood with Satterthwaite degrees of freedom. All available outcome measurements were included for patients with complete data for the adjustment variables. Additional exploratory models incorporated available measurements from T0, T1, and T2.

All tests were two-sided, with a nominal *P* < 0.05 considered statistically significant. No adjustment for multiple comparisons was performed, and all reported *P* values are nominal. Analyses of clinical outcomes and biomarkers were exploratory. Analyses were performed using IBM SPSS Statistics version 26 and R software.

## 3 Results

### 3.1 Baseline characteristics

A total of 72 patients with moderate-to-severe asthma were included, comprising 48 receiving OMA and 24 receiving DUP (Figure 1). Patients receiving OMA were younger than those receiving DUP (37 ± 12 vs. 45 ± 12 years, *P* = 0.011). Atopic dermatitis was more frequent in the DUP group (62.5% vs. 14.6%, *P* < 0.001). No statistically significant between-group differences were detected in sex, BMI, asthma duration, baseline ACT score, maintenance systemic corticosteroid use, BEC, serum total IgE, FeNO, or pulmonary function indices (Table 1).

**Figure 1:**
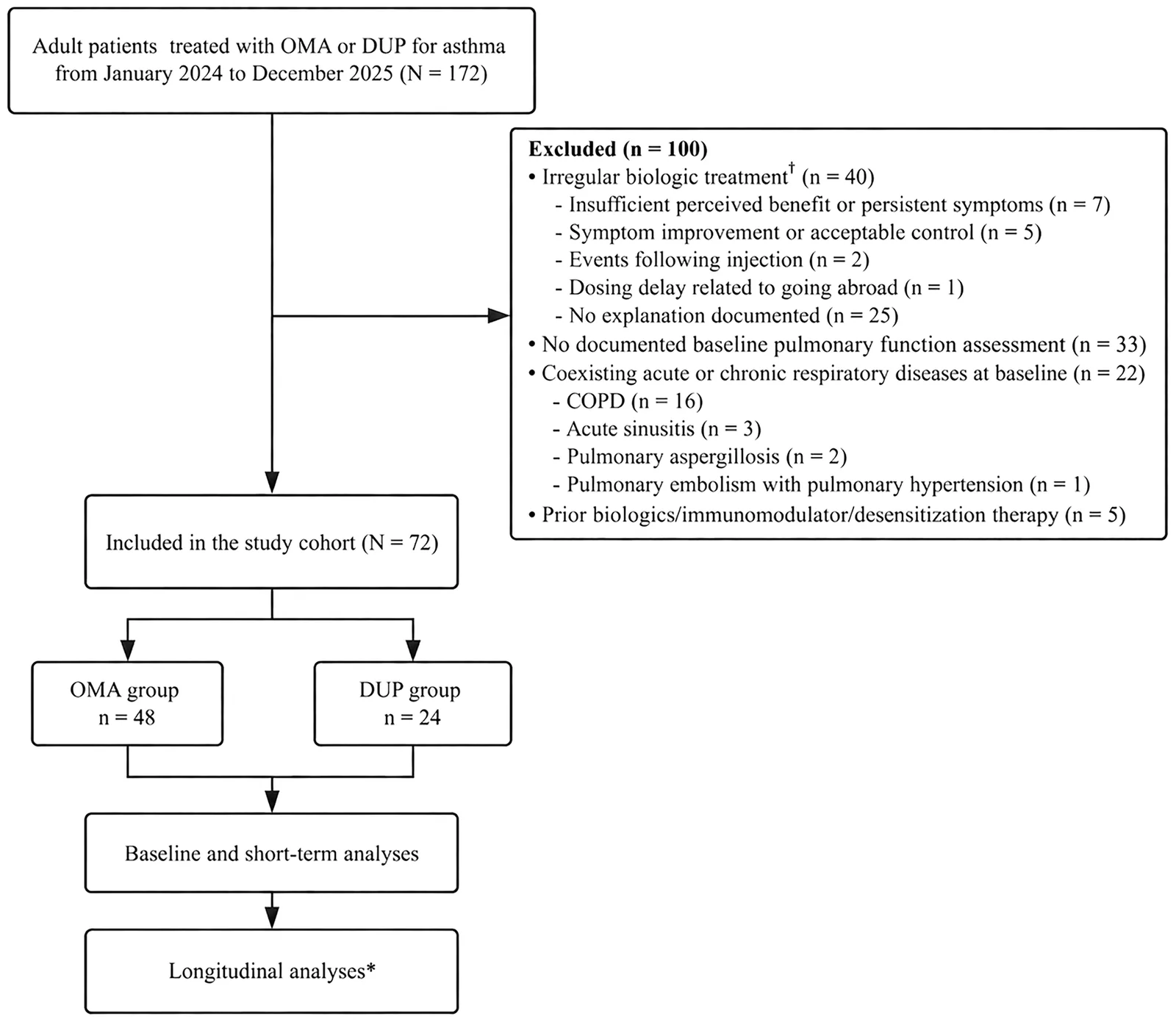
Flow diagram of patient screening and study inclusion. OMA, Omalizumab; DUP, Dupilumab; COPD, chronic obstructive pulmonary disease. ^†^Recorded circumstances were not consistently documented as reasons for treatment interruption. *Linear mixed-effects models incorporated all available repeated measurements and did not require complete follow-up data. Twenty-nine patients contributed T2 assessments while remaining on their index biologic (OMA, *n* = 23; DUP, *n* = 6); availability varied by outcome.

**Table 1:** Baseline characteristics of patients receiving omalizumab or dupilumab.

Characteristic	All (*n* = 72)	Omalizumab (*n* = 48)	Dupilumab (*n* = 24)	*P* value
Age, years, mean ± SD	39 ± 12	37 ± 12	45 ± 12	**0**.**011**
Female, *n* (%)	44 (61.11%)	31 (64.58%)	13 (54.17%)	0.393
BMI, kg/m^2^, mean ± SD	22.68 ± 3.12	22.41 ± 3.34	23.24 ± 2.61	0.292
Asthma duration, years, median (IQR)	1.50 (0.83, 4)	1.58 (0.90, 3)	1.25 (0.42, 6)	0.850
ACT score, median (IQR)	21 (18.75, 22)	21 (18, 22)	21.5 (19, 22)	0.522
Maintenance SCS use, *n* (%)	6 (8.33%)	3 (6.25%)	3 (12.50%)	0.651
BEC, ×10^9^/L, median (IQR)	0.24 (0.13, 0.47)	0.27 (0.16, 0.52)	0.20 (0.08, 0.35)	0.117
Total IgE, IU/mL, median (IQR)	188 (113.75, 463)	203.5 (122.25, 543.75)	161.5 (67.4, 254.75)	0.134
Aeroallergen sensitization^a^, *n* (%)	54 (79.41%)	44 (93.62%)	10 (47.62%)	**<0**.**001**
Dust mite sensitization^a^, *n* (%)	30 (44.12%)	25 (53.19%)	5 (23.81%)	**0**.**034**
Animal dander sensitization^a^, *n* (%)	30 (44.12%)	26 (55.32%)	4 (19.05%)	**0**.**008**
FeNO_50_^b^, ppb, median (IQR)	37 (21, 93)	38 (26.5, 92.5)	31 (18.5, 90)	0.585
FEV_1_^c^, L, median (IQR)	2.72 (2.18, 3.44)	2.79 (2.18, 3.64)	2.68 (2.26, 2.97)	0.337
ppFEV_1_^c^, %, mean ± SD	89.11 ± 17.82	91.01 ± 16.88	85.40 ± 19.38	0.222
FEV_1_/FVC^c^, %, mean ± SD	76.02 ± 10.51	77.36 ± 10.06	73.40 ± 11.09	0.144
FEF_25–75_^c^, L/s, median (IQR)	2.22 (1.62, 3.39)	2.54 (1.71, 3.71)	1.83 (1.21, 2.92)	0.125
Percent-predicted FEF_25−75_^c^, %, mean ± SD	63.04 ± 27.81	66.42 ± 27.18	56.41 ± 28.45	0.162
Allergic rhinitis, *n* (%)	65 (90.28%)	44 (91.67%)	21 (87.50%)	0.888
CRSwNP, *n* (%)	3 (4.17%)	2 (4.17%)	1 (4.17%)	1.000
Atopic dermatitis, *n* (%)	22 (30.56%)	7 (14.58%)	15 (62.50%)	**<0.001**
Urticaria, *n* (%)	20 (27.78%)	16 (33.33%)	4 (16.67%)	0.137

Bold *P* values indicate nominal statistical significance (*P* < 0.05).

a Available serum allergen-specific IgE results: OMA, *n* = 47; DUP, *n* = 21.

b Available FeNO_50_ results: OMA, *n* = 32; DUP, *n* = 19.

c Available numerical pulmonary function results: OMA, *n* = 45; DUP, *n* = 23.

OMA, omalizumab; DUP, dupilumab; BMI, body mass index; ACT, Asthma Control Test; SCS, systemic corticosteroid; BEC, blood eosinophil count; IgE, immunoglobulin E; FeNO_50_, fractional exhaled nitric oxide at 50 mL/s; FEV_1_, forced expiratory volume in one second; ppFEV_1_, percent-predicted FEV_1_; FVC, forced vital capacity; FEF_25–75_, forced expiratory flow at 25%–75% of FVC; CRSwNP, chronic rhinosinusitis with nasal polyps; SD, standard deviation; IQR, interquartile range..

Recent pretreatment serum aeroallergen-specific IgE results were available for 47 OMA-treated and 21 DUP-treated patients. Aeroallergen sensitization was detected in 44/47 (93.62%) and 10/21 (47.62%), respectively (*P* < 0.001). The three OMA-treated patients with negative results had a history of pollen or cat allergy. The remaining OMA-treated patient declined pretreatment testing and had elevated dust mite-specific IgE after treatment initiation. All four received OMA for clinically diagnosed allergic asthma and had no concomitant chronic urticaria.

Dust mite sensitization was present in 25/47 OMA-treated and 5/21 DUP-treated patients (53.19% vs. 23.81%, *P* = 0.034). Animal dander sensitization was present in 26/47 and 4/21 patients, respectively (55.32% vs. 19.05%, *P* = 0.008). Among aeroallergen-sensitized patients, neither dust mite sensitization (25/44 vs. 5/10, *P* = 0.736) nor animal dander sensitization (26/44 vs. 4/10, *P* = 0.311) differed significantly between groups. The overall distribution of sensitization profiles also did not differ significantly (exact *P* = 0.697; Supplementary Figure S1).

### 3.2 Changes from baseline to 4 months

Short-term comparisons used the available paired observations for each outcome rather than requiring complete data across all outcomes. Paired ACT scores were available for 48 OMA-treated and 24 DUP-treated patients, paired ppFEV_1_ measurements for 25 and 14 patients, and paired total IgE measurements for 27 and 11 patients, respectively. Outcome-specific denominators for the remaining measurements are provided in Table 2.

**Table 2:** Changes in clinical, inflammatory, and pulmonary function parameters from baseline to 4 months.

Outcome	Omalizumab (*n* = 48)			Dupilumab (*n* = 24)		
	T0	T1	*P* value	T0	T1	*P* value
ACT score^a^, median (IQR)	21 (18, 22)	24 (23, 25)	**<0.001**	21.5 (19, 22)	24.5 (23, 25)	**<0.001**
BEC^b^, ×10^9^/L, median (IQR)	0.29 (0.17, 0.49)	0.15 (0.07, 0.31)	**0**.**005**	0.14 (0.07, 0.22)	0.10 (0.06, 0.15)	0.326
Total IgE^c^, IU/mL, median (IQR)	176 (113.5, 500)	328 (202, 812)	**<0.001**	133 (31.55, 307)	48.8 (14.25, 137)	**0**.**002**
FeNO_50_^d^, ppb, median (IQR)	56 (29, 104.75)	25.5 (23, 36.75)	**<0.001**	30 (19, 63)	23 (16, 33)	**0**.**027**
FeNO_200_^e^, ppb, mean ± SD	21.87 ± 15.39	12.67 ± 8.46	**0**.**008**	16.33 ± 12.94	11 ± 4.73	0.344
FnNO^e^, ppb, mean ± SD	731.53 ± 421.52	630.93 ± 219.91	0.191	711.33 ± 208.72	672.83 ± 295.22	0.825
CaNO^e^, ppb, mean ± SD	5.01 ± 3.90	3.17 ± 2.65	0.178	3.75 ± 2.44	3.10 ± 2.81	0.731
FEV_1_^f^, L, median (IQR)	2.53 (2.16, 2.84)	2.71 (2.47, 3.20)	**0**.**004**	2.62 (2.40, 2.82)	2.77 (2.51, 3.07)	**0**.**025**
ppFEV_1_^f^, %, median (IQR)	85.32 (77.37, 94.60)	93.84 (87.60, 101.57)	**0**.**001**	83.89 (78.21, 94.56)	90.97 (84.53, 100.79)	**0**.**020**
FEV_1_/FVC^f^, %, median (IQR)	73.08 (66.59, 82.16)	81.15 (73.95, 86.33)	**<0.001**	72.42 (67.76, 73.44)	75.37 (71.80, 82.30)	**0**.**007**
FEF_25−75_^f^, L/s, median (IQR)	2.05 (1.11, 2.69)	2.70 (2.19, 3.56)	**<0.001**	1.71 (1.19, 2.15)	2.17 (1.63, 3.16)	**0**.**012**
Percent-predicted FEF_25–75_^f^, %, median (IQR)	52.04 (37.41, 75.00)	71.47 (55.29, 94.10)	**0**.**001**	43.80 (36.71, 56.12)	62.71 (48.91, 85.69)	**0**.**008**

Paired *n* (OMA/DUP):

a 48/24.

b 28/14.

c 27/11.

d 18/13.

e 15/6.

f 25/14.

*P* values indicate within-group T0–T1 comparisons. Bold *P* values indicate nominal statistical significance (*P* < 0.05).

OMA, omalizumab; DUP, dupilumab; ACT, Asthma Control Test; BEC, blood eosinophil count; IgE, immunoglobulin E; FeNO_50_/_200_, fractional exhaled nitric oxide at 50/200 mL/s; FnNO, fractional nasal nitric oxide; CaNO, alveolar nitric oxide; FEV_1_, forced expiratory volume in one second; ppFEV_1_, percent-predicted FEV_1_; FVC, forced vital capacity; FEF_25–75_, forced expiratory flow at 25%–75% of FVC; SD, standard deviation; IQR, interquartile range.

All patients remained on their index biologic through T1. Before T1, no OMA-treated patient had a documented dosing delay, interval extension, early discontinuation, or biologic switch (0/48 for each). One DUP-treated patient temporarily extended the dosing interval before T1 (1/24, 4.2%) but remained on DUP through the assessment; no other dosing delays, early discontinuations, or switches occurred in this group before T1. This patient subsequently switched to benralizumab after T1, and only observations obtained before switching were included.

ACT scores increased from baseline in both treatment groups (both *P* < 0.001). ppFEV_1_ also increased in the OMA group (*P* = 0.001) and the DUP group (*P* = 0.020). Improvements were additionally observed in FEV_1_, FEV_1_/FVC, forced expiratory flow at 25%–75% of forced vital capacity (FEF_25–75_), and percent-predicted FEF_25–75_ in each group (all *P* < 0.05). In the OMA group, BEC, FeNO_50_, and FeNO at 200 mL/s (FeNO_200_) decreased, whereas total IgE increased; fractional nasal nitric oxide (FnNO) and alveolar nitric oxide (CaNO) did not change significantly. In the DUP group, total IgE and FeNO_50_ decreased, while no statistically significant changes in BEC, FeNO_200_, FnNO, or CaNO were detected (Table 2).

Between-group comparisons did not detect differences in changes in ACT score, ppFEV_1_, BEC, FeNO, or the other pulmonary function indices. The change in total IgE differed between groups (*P* < 0.001), reflecting an increase after OMA and a decrease after DUP (Figure 2).

**Figure 2:**
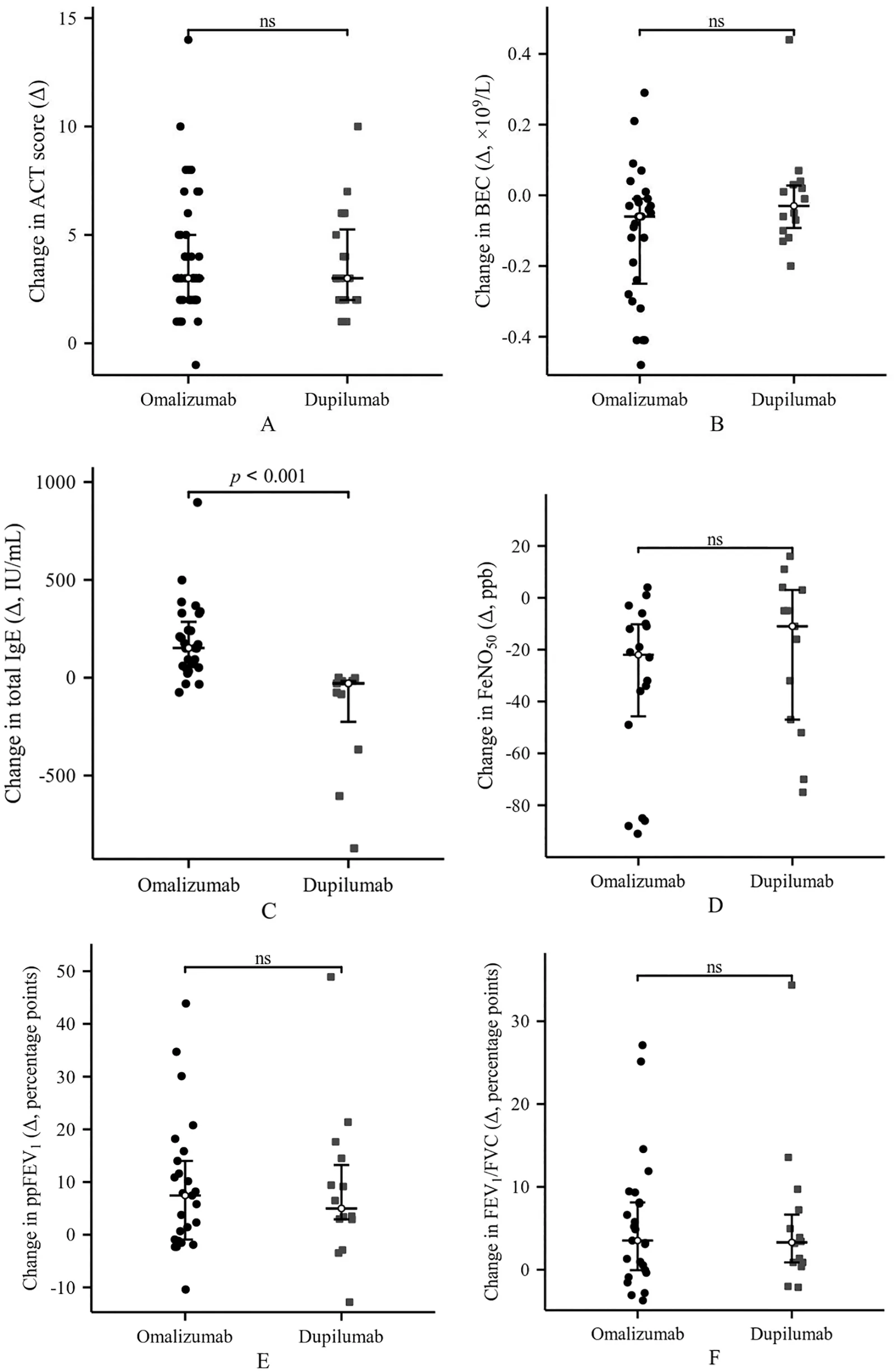
Changes in clinical, inflammatory, and pulmonary function parameters from baseline to 4 months. Changes were calculated as Δ = T1–T0. Symbols represent individual patients; central horizontal lines indicate medians, and error bars extend from the 25th to the 75th percentile. Between-group comparisons used two-sided Mann–Whitney *U*-tests. OMA, omalizumab; DUP, dupilumab; ACT, Asthma Control Test; BEC, blood eosinophil count; IgE, immunoglobulin E; FeNO_50_, fractional exhaled nitric oxide at 50 mL/s; ppFEV_1_, percent-predicted FEV_1_; FVC, forced vital capacity; ns, not statistically significant.

Within the included cohort, adverse events were documented in three OMA-treated patients (6.3%) and included injection-site swelling and transient urticaria-like reactions. No adverse events were documented in the DUP group, and no severe hypersensitivity reactions or treatment-related deaths occurred.

Maintenance systemic corticosteroid use was recorded in three patients in each group at T0. No maintenance systemic corticosteroid use was recorded at T1 or among patients assessed at T2 in either group.

### 3.3 Adjusted analyses and extended follow-up

ACT scores were available at T0, T1, and T2 for 48, 48, and 23 OMA-treated patients and 24, 24, and 6 DUP-treated patients, respectively. Corresponding availability was 45, 27, and 13 vs. 23, 15, and 3 for ppFEV_1_ and 48, 27, and 10 vs. 24, 11, and 1 for total IgE. Missing pulmonary function and biomarker values mainly reflected tests that were not repeated or whose results were unavailable during follow-up.

Among the 43 patients without on-treatment T2 data, 19 OMA-treated and 15 DUP-treated patients had discontinued the index biologic, while 6 and 3 patients, respectively, had switched biologics. Documented reasons for switching mainly included insufficient perceived benefit or persistent comorbid symptoms. Reasons for patient-initiated discontinuation were not consistently documented.

After excluding four patients without aeroallergen sensitization data, the adjusted T0–T1 models included 68 patients and 136 observations for ACT, 67 patients and 105 observations for ppFEV_1_, and 68 patients and 105 observations for ln(total IgE). ACT score and ppFEV_1_ increased over time (*P* < 0.001 and *P* = 0.002, respectively), without significant treatment-group effects (*P* = 0.819 and *P* = 0.694) or treatment-by-time interactions (*P* = 0.591 and *P* = 0.931). For ln(total IgE), neither the treatment-group effect (*P* = 0.223) nor the time effect (*P* = 0.988) was significant, whereas the treatment-by-time interaction was significant (*P* < 0.001), indicating different four-month changes between groups (Table 3).

**Table 3:** Adjusted linear mixed-effects models of changes from baseline to 4 months.

Outcome	Treatment-group effect, *P* value	Time effect, *P* value	Treatment-by-time interaction, *P* value
ACT score	0.819	**<0** **.** **001**	0.591
ppFEV_1_	0.694	**0** **.** **002**	0.931
ln (total IgE)	0.223	0.988	**<0** **.** **001**

Bold *P* values indicate nominal statistical significance (*P* < 0.05).

ACT, Asthma Control Test; ppFEV_1_, percent-predicted forced expiratory volume in one second; IgE, immunoglobulin E.

Exploratory models incorporating T0, T1, and T2 included 68 patients and 164 observations for ACT, 67 patients and 120 observations for ppFEV_1_, and 68 patients and 116 observations for ln(total IgE). No significant treatment-by-time interactions were detected for ACT (*P* = 0.842) or ppFEV_1_ (*P* = 0.683), whereas the interaction for ln(total IgE) was significant (*P* < 0.001; Supplementary Table S1).

### 3.4 Assessment of the clinical overlap population

Using pretreatment eligibility information, 41 of the 72 patients met the stringent definition of clinical eligibility for either treatment, including 33 receiving OMA and 8 receiving DUP. Baseline characteristics are summarized descriptively in Supplementary Table S2. Because the treatment groups were small and markedly unbalanced and paired follow-up measurements were sparse, particularly in the DUP group, a formal restricted comparison of outcomes was not performed.

## 4 Discussion

In this retrospective, single-center, real-world cohort, treatment with either OMA or DUP was associated with improved asthma control and pulmonary function. No significant between-group differences in clinical changes were detected, whereas serum total IgE showed opposing changes over the first 4 months. These findings describe patients who continued treatment through T1 under routine clinical selection.

Patients receiving OMA were younger and more frequently sensitized to aeroallergens, whereas atopic dermatitis was more common among those receiving DUP. These differences are consistent with phenotype- and comorbidity-guided prescribing in routine practice (9, 13). Previous real-world studies have similarly reported preferential use of DUP in patients with concomitant atopic dermatitis (16, 17). Treatment accessibility may also influence clinical selection. Adjustment for age, sex, atopic dermatitis, and overall aeroallergen sensitization helped account for these baseline differences, although residual confounding remains possible.

We also identified a stringent clinical overlap population using pretreatment eligibility information. Only 41 patients met the concurrent criteria, including 33 treated with OMA and 8 treated with DUP. Because the groups were small and markedly unbalanced and paired follow-up measurements were unevenly available, their baseline characteristics were reported descriptively and a formal restricted outcome comparison was not undertaken. The inability to obtain reliable comparative estimates within this population further limits treatment-specific interpretation.

ACT score and ppFEV_1_ improved from baseline to 4 months in the adjusted models, with no significant treatment-group effects or treatment-by-time interactions. The exploratory models incorporating available 12-month measurements showed a similar overall pattern, although longer-term comparisons were limited by sparse follow-up. These findings are consistent with previous studies reporting improvements in asthma control and pulmonary function during OMA or DUP treatment (18–21). Some comparative studies have reported larger improvements with DUP than with OMA (11, 12); differences in population characteristics, prescribing practices, outcome definitions, and follow-up duration may contribute to the variation across studies.

The clearest between-treatment difference at 4 months was observed in serum total IgE. OMA was associated with an increase in total IgE after treatment initiation, whereas DUP was associated with a decrease, resulting in a significant treatment-by-time interaction. This divergence is biologically plausible given the distinct mechanisms of the two agents. OMA forms IgE–anti-IgE complexes, which can increase measured total IgE despite reducing free IgE activity (22). In contrast, blockade of IL-4/IL-13 signaling by DUP can suppress IgE class switching and production (3, 23). Reductions in type 2 inflammatory biomarkers during DUP treatment have also been reported previously (24). The opposing 4-month changes and the interaction in the T0–T1 analysis are consistent with biologic-specific pharmacodynamic effects, although residual confounding remains possible. With only one DUP-treated patient contributing a total IgE measurement at T2, the available data were insufficient to characterize a reliable 12-month IgE trajectory in this group. We did not assess whether IgE changes predicted clinical improvement or could guide treatment monitoring or biologic selection.

Among patients included in the cohort, few adverse events were documented, and no severe hypersensitivity reactions or treatment-related deaths occurred. The observed events were consistent with the established safety profiles of these treatments (25–27). However, the sample size and follow-up duration were insufficient for a reliable comparative safety assessment or detection of uncommon adverse events.

Several limitations should be acknowledged. First, the retrospective, single-center design and modest, unequal group sizes limited precision and generalizability. Second, non-random allocation and restriction to four-month completers may bias effectiveness and safety estimates. Residual confounding by specific sensitization profiles remains possible despite adjustment, and the small, unbalanced overlap population precluded reliable restricted comparisons. Third, incomplete measurements, particularly at T2, limited interpretation, and informative missingness cannot be excluded. Fourth, exacerbations and changes in background asthma therapy and topical treatment for atopic dermatitis were incompletely documented. Finally, multiple outcomes were tested without multiplicity adjustment, increasing the risk of false-positive findings; these exploratory results require confirmation.

## 5 Conclusion

In this cohort, OMA and DUP were each associated with improvements in asthma control and pulmonary function, with no clear between-group differences detected. Serum total IgE showed opposing 4-month changes that were consistent with the distinct pharmacodynamic effects of the two treatments. Larger prospective multicenter studies with more complete follow-up and standardized outcome collection are warranted to further validate these findings.

## Data Availability

The original contributions presented in the study are included in the article/Supplementary Material, further inquiries can be directed to the corresponding author.
