# Supplementary material for: Clinical outcomes and total IgE changes with omalizumab and dupilumab in moderate-to-severe asthma: a retrospective real-world cohort study
Source: Front Med (Lausanne). 2026 Oct 20;13:1964856. doi: 10.3389/fmed.2026.1964856 (PMC13727635; doi:10.3389/fmed.2026.1964856)
Supplement: Supplementary file 1 [file Presentation1.pdf]

Supplementary Material

Clinical outcomes and total IgE changes with omalizumab and dupilumab in moderate-to-severe asthma: a retrospective real-world cohort study

Figure S1. Distribution of aeroallergen sensitization profiles by biologic treatment group.

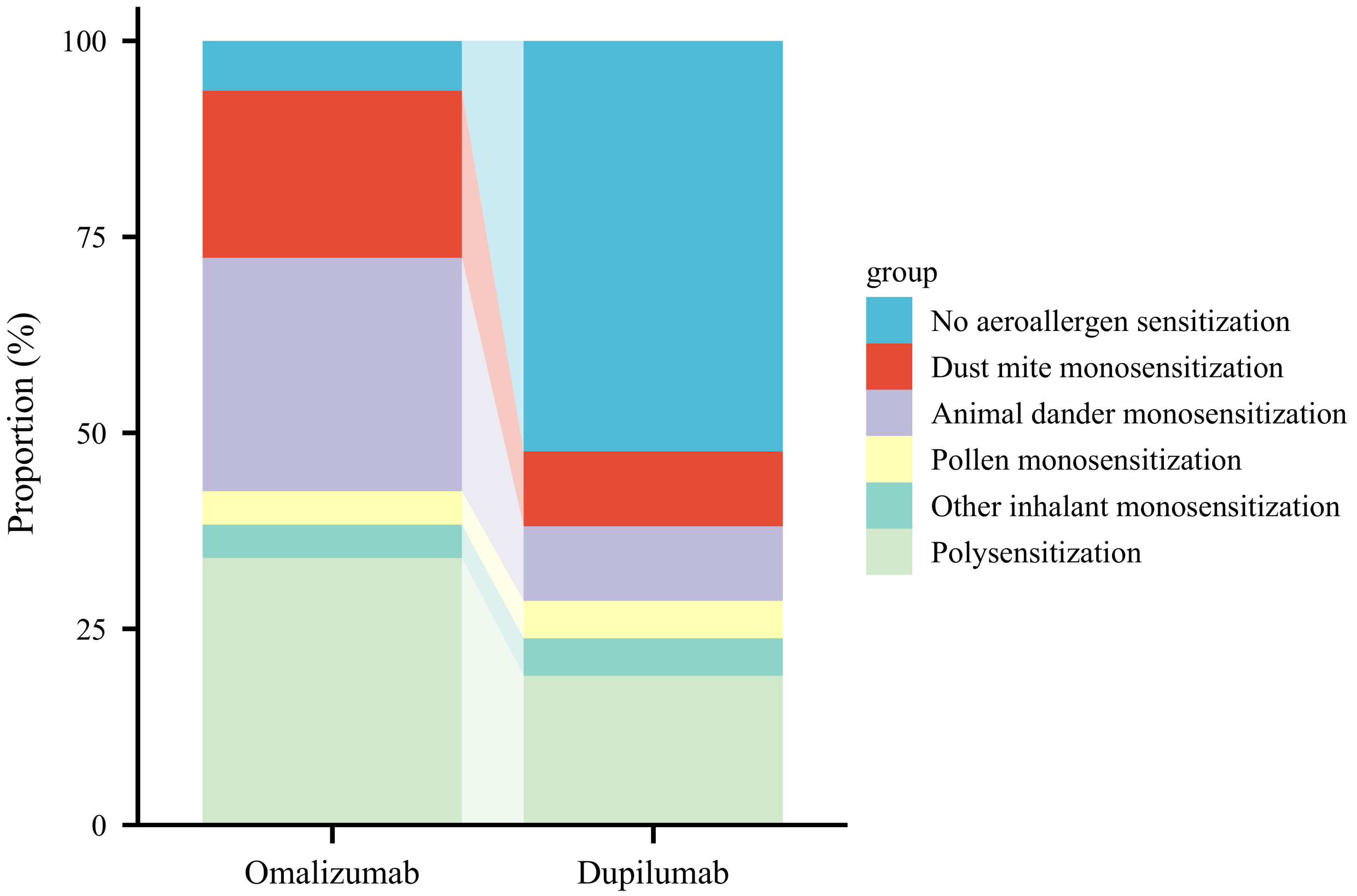

Percentages were calculated among patients with available pretreatment testing (OMA, n = 47; DUP, n = 21). Polysensitization was defined as sensitization to ≥2 aeroallergen categories.

Abbreviations: OMA, omalizumab; DUP, dupilumab.

Supplementary Table S1. Exploratory adjusted linear mixed-effects models incorporating baseline, 4-month, and 12-month measurements.

Outcome	Treatment-group effect P value	Time effect P value	Treatment-by-time interaction P value
ACT score	0.739	**< 0.001**	0.842
$ppFEV_1$	0.881	**0.002**	0.683
ln (total IgE)	0.078	0.338	**< 0.001**

Bold P values indicate nominal statistical significance (P < 0.05).

Abbreviations: ACT, Asthma Control Test; $ppFEV_1$, percent-predicted forced expiratory volume in one second; IgE, immunoglobulin E.

Supplementary Table S2. Baseline characteristics of the study-defined overlap population.

Characteristic	All (n = 41)	Omalizumab (n = 33)	Dupilumab (n = 8)
Age, years, mean ± SD	35 ± 11	35 ± 11	37 ± 8
Female, n (%)	26 (63.4%)	21 (63.6%)	5 (62.5%)
BMI, kg/m², mean ± SD	21.65 ± 2.67	21.45 ± 2.64	22.49 ± 2.81
Asthma duration, years, median (IQR)	1.50 (0.67, 3)	1.67 (0.92, 3)	0.375 (0.21, 7.60)
ACT score, median (IQR)	21 (18, 22)	21 (18, 22)	22 (21.75, 22.25)
Maintenance SCS use, n (%)	3 (7.3%)	2 (6.1%)	1 (12.5%)
BEC, $\times 10^9$/L, median (IQR)	0.34 (0.22, 0.52)	0.34 (0.24, 0.52)	0.24 (0.21, 0.35)
BEC ≥ 0.15×10^9/L, n (%)	38 (92.7%)	30 (90.9%)	8 (100%)
Total IgE, IU/mL, median (IQR)	279 (140, 693)	322 (131, 693)	208.5 (162.75, 682.5)
$FeNO_{50}$†, ppb, median (IQR)	56 (29, 94)	52 (29, 94)	63 (18, 90)
$FeNO_{50}$ ≥ 25 ppb†, n (%)	23 (82.1%)	19 (90.5%)	4 (57.1%)
FEV_1‡, L, median (IQR)	2.85 (2.29, 3.64)	2.82 (2.22, 3.76)	3.09 (2.77, 3.53)
$ppFEV_1$‡, %, mean ± SD	90.67 ± 20.67	91.57 ± 19.75	86.67 ± 25.72
FEV_1/FVC‡, %, median (IQR)	79.21 (71.10, 83.97)	78.24 (70.93, 83.46)	84.05 (78.74, 85.85)
FEF_{25-75}‡, L/s, mean ± SD	2.81 ± 1.42	2.73 ± 1.44	3.14 ± 1.40
Percent-predicted FEF_{25-75}‡, %, mean ± SD	68.67 ± 31.88	66.68 ± 31.25	77.47 ± 35.66
Allergic rhinitis, n (%)	37 (90.2%)	30 (90.9%)	7 (87.5%)
CRSwNP, n (%)	2 (4.9%)	2 (6.1%)	0 (0%)
Atopic dermatitis, n (%)	10 (24.4%)	3 (9.1%)	7 (87.5%)
Urticaria, n (%)	14 (34.1%)	12 (36.4%)	2 (25%)

† Available $FeNO_{50}$ results: OMA, n = 21; DUP, n = 7.

‡ Available numerical pulmonary function results: OMA, n = 31; DUP, n = 7.

Abbreviations: BMI, body mass index; ACT, Asthma Control Test; SCS, systemic corticosteroid; BEC, blood eosinophil count; IgE, immunoglobulin E; $FeNO_{50}$, fractional exhaled nitric oxide at 50 mL/s; FEV_1, forced expiratory volume in one second; $ppFEV_1$, percent-predicted FEV_1; FVC, forced vital capacity; FEF_{25-75}, forced expiratory flow at 25-75% of FVC; CRSwNP, chronic rhinosinusitis with nasal polyps; SD, standard deviation; IQR, interquartile range.
